# Hospitalization and Successful Aging of Community-Dwelling Older Adults: Using the Korean National Survey Data

**DOI:** 10.1093/geroni/igaa057.451

**Published:** 2020-12-16

**Authors:** Ji Yeon Lee, Bora Kim, Kyung Hee Lee, Changgi Park

**Affiliations:** 1 Yonsei University, Seoul, Republic of Korea; 2 Yonsei University College of Nursing, Seoul, Republic of Korea; 3 University of Illinois, Chicago, Illinois, United States

## Abstract

Hospitalization experience can be an obstructive factor to successful aging. Although older adults who had hospitalization experience has been considered to have poor health status and low participation in one’s life, it is not obviously evident whether hospitalization itself affects successful aging. This study aimed to investigate whether three components of successful aging (i.e., diseases and disease-related complications, physical and mental functions, and engagement with life) were different in community-dwelling older adults who had hospitalization experience for the past one year compared to the counterpart older adults without hospitalization experience. A secondary data analysis was performed using a nationally representative survey data in Korea. A total of 1,812 who had hospitalization experience were matched to 1,812 control counterpart using propensity score matching. Sampling weight of the survey was considered for all statistical analysis. The community-dwelling older adults with hospitalization experience were less likely to be aging successfully than the older adults without hospitalization experience. The older adults with hospitalization experience had more chronic illnesses and malnourishment; they had more impairment in physical function and depressive symptoms; they were less active in working, social activities, and traveling. However, there were no differences in cognitive function and religious activities between the groups. In conclusion, the community-dwelling older adults who had hospitalization experience have poor health status and less engagement in one’s life in general after matching covariates using propensity score matching analysis. Therefore, more attention and assist are needed to the community-dwelling older adults with hospitalization experience to facilitate successful aging.

